# Documenting First Nations Access to COVID Vaccines: A whole-population linked administrative data study.

**DOI:** 10.23889/ijpds.v7i3.1947

**Published:** 2022-08-25

**Authors:** Nathan C. Nickel, Wanda Phillips-Beck, Leona Star, Ekuma Okechukwu, Carole Taylor, Oludolapo Deborah Balogun, Marni Brownell, Mariette Chartier, Dan Chateau, Jennifer Enns, Alan Katz, Josee Lavoie, Lisa Lix, Alyson Mahar, Razvan Romanescu, Marcelo Urquia

**Affiliations:** 1 Manitoba Centre for Health Policy, University of Manitoba; 2 First Nations Health and Social Secretariat of Manitoba; 3 MCHP; 4 University of Manitoba; 5 Australian National University; 6 Ongomiizwin, University of Manitoba

## Objectives

First Nations (FN) organizations worked with public health and governments to improve FN access to COVID-19 vaccines by prioritizing FN communities in vaccination initiatives. FN researchers and data scientists partnered to test whether these efforts were associated with increased access to COVID-19 vaccines among FN compared with all other Manitobans.

## Approach

This retrospective cohort study linked whole-population administrative data from (i) the First Nations research file, (ii) COVID testing and vaccination data, and (iii) health and social services for sociodemographic data and information on potential confounders. Several public health policies were created to improve access to COVID vaccines among FN; we tested whether FN received their 1st and 2nd vaccines sooner than all other Manitobans (AOM) using restricted mean survival time models. We adjusted for sociodemographic characteristics, comorbidities, and whether FN lived on- or off-reserve. We conducted sex-specific and effect modification analyses to test whether associations differed by sex.

## Results

Prioritizing FN to receive vaccines was associated with increased vaccine uptake compared with AOM. After adjusting for various confounders, FN received their first dose 15.5 (95% CI 14.9 – 16.0) days sooner than AOM and their second dose 13.9 (13.3 – 14.5) days sooner than AOM. Sex-stratified and subsequent effect modification analyses using interaction terms, found that differences were greater for males than for females: FN males received their first dose 18.1 (17.3 – 18.8) days sooner than AOM males and FN females received their first dose 12.9 (12.2 – 13.7) days sooner than AOM females. This pattern held for second doses as well. FN with comorbidities also received vaccines sooner than AOM with similar comorbidity levels 20.9 days (23.1 – 18.8) among those with 3+ comorbidities.

## Conclusion

Partnerships between public health entities and FN organizations that respect FN community sovereignty were instrumental in supporting FN health and well-being during COVID-19. Policies and programs that prioritized FN people for vaccines improved uptake saving lives. This partnership-based COVID-19 response can provide a framework for future public health efforts.

